# Individuals motivated to participate in adherence, care and treatment (imPACT): development of a multi-component intervention to help HIV-infected recently incarcerated individuals link and adhere to HIV care

**DOI:** 10.1186/s12889-016-3511-1

**Published:** 2016-09-06

**Authors:** Carol E. Golin, Kevin Knight, Jessica Carda-Auten, Michele Gould, Jennifer Groves, Becky L.White, Steve Bradley-Bull, Kemi Amola, Niasha Fray, David L. Rosen, Michael J. Mugavaro, Brian W. Pence, Patrick M. Flynn, David Wohl

**Affiliations:** 1 School of Medicine and Gillings School of Global Public Health, The University of North Carolina at Chapel Hill, Chapel Hill, NC 27599 USA; 2 Institute of Behavioral Research, Texas Christian University, Fort Worth, USA; 3 School of Medicine, The University of North Carolina at Chapel Hill, Chapel Hill, USA; 4 Cecil G. Sheps Center, The University of North Carolina at Chapel Hill, Chapel Hill, USA; 5 Gillings School of Global Public Health, The University of North Carolina at Chapel Hill, Chapel Hill, USA; 6 The University of Alabama at Birmingham, Birmingham, USA; 7 School of Medicine, 321 S The University of North Carolina at Chapel Hill, Chapel Hill, USA; 8 Department of Health Behavior, UNC-CH Gillings School of Global Public, CB 7440, 135 Dauer Road, Chapel Hill, NC 27599 USA

**Keywords:** HIV, Medication adherence, Retention in care, Justice-involved individuals

## Abstract

**Background:**

Policy-makers promote a seek, test, treat and retain (STTR) strategy to expand HIV testing, support linkage and engagement in care, and enhance the continuous use of antiretroviral therapy for those HIV-infected. This HIV prevention strategy is particularly appropriate in correctional settings where HIV screening and treatment are routinely available yet many HIV-infected individuals have difficulty sustaining sufficient linkage and engagement in care, disease management, and viral suppression after prison release.

**Methods/design:**

Our research team developed Project imPACT (individuals motivated to Participate in Adherence, Care and Treatment), a multi-component approach for HIV-Infected recently incarcerated individuals that specifically targets their care linkage, retention, and medication adherence by addressing multiple barriers to care engagement after release. The ultimate goals of this intervention are to improve the health of HIV-infected individuals recently released from prison and reduce HIV transmission to their communities by maintaining viral suppression. This paper describes the intervention and technology development processes, based on best practices for intervention development and process evaluation. These processes included: 1) identifying the target population; 2) clarifying the theoretical basis for intervention design; 3) describing features of its foundational interventions; 4) conducting formative qualitative research; 5) integrating and adapting foundational interventions to create and refine intervention content based on target audience feedback. These stages along with the final intervention product are described in detail. The intervention is currently being evaluation and a two arm randomized, controlled trial in two US state prison systems.

**Discussion:**

Based on a literature review, qualitative research, integration of proven interventions and behavioral theory, the final imPACT intervention focused on the transition period two to three months before and three months after prison release. It emphasized pre-release readiness, pre- and post-release supportive non-judgmental counseling, linking individuals to a HIV care clinic and technological supports through videos and text messages. This article provides a useful model for how researchers can develop, test, and refine multi-component interventions to address HIV care linkage, retention and adherence.

**Clinical trial registration:**

NCT01629316, first registered 6-4-2012; last updated 6-9-2015.

## Background

It is now widely recognized that individuals who maintain an undetectable plasma HIV-1 RNA (viral load) can live a healthy, nearly normal life span and have markedly reduced risk of transmitting HIV to other individuals [1–5]. Theoretically, achieving timely diagnosis, linkage and retention in care, and appropriate HIV treatment among all HIV-infected persons could substantially reduce--if not eliminate--the HIV epidemic [6–8]. The continued occurrence of 40,000 to 50,000 new HIV infections annually in the United States [9], despite the availability of effective treatment and prevention methods, has prompted researchers and policy-makers to investigate gaps in implementation. Using what has been termed “the HIV treatment cascade,” researchers found that high proportions of HIV-infected individuals drop off at each of several key steps, with the largest, an approximately 50 % drop-off, occurring between diagnosis with HIV and consistent engagement in care [10]. These observations have led policy-makers to promote a seek, test, treat and retain (STTR) strategy to expand HIV testing, support linkage and engagement in care, and enhance the continuous use of antiretroviral therapy (ART) for those identified as HIV-infected. In fact, the US Centers for Disease Control and Prevention Division of HIV AIDS Prevention and the US National AIDS Strategy [11] emphasizes increasing testing, linkage and retention in care [10, 12, 13] as a means to prevent HIV transmission.

The STTR approach to HIV prevention is particularly appropriate among certain high risk groups, such as those in correctional settings. HIV screening is available and conducted routinely in prison within the United States. HIV prevalence among incarcerated persons is three to five times higher than that of the general population [14–16]. In studies conducted in Texas and North Carolina, respectively, between 2004 and 2009, about 55 to 59 % of HIV-infected inmates leave prison with suppressed viral loads [17, 18] although in a 2010 study, Baillargeon and colleagues reported only 37 % of HIV-infected releasees with an undetectable viral load at prison release [14].

While many HIV-infected individuals are diagnosed with HIV and receive recommended HIV medical treatment in prison, most have difficulty sustaining sufficient linkage and engagement in care, disease management, and viral suppression after prison release. In one study, only 30 % of HIV-infected released individuals had filled their antiretroviral prescriptions within 60 days of release [17]. In other studies of care engagement, only 20 to 54 % of HIV-infected individuals released from prison had enrolled in an HIV clinic within one month of release [14, 19]. Not surprisingly, viral loads increase after release from prison [18, 20]. Moreover, as expected, such disruptions in care result in higher HIV-associated morbidity, mortality, and viral resistance to ART in recently released individuals [21–23] and raises the potential for transmission of the virus. Given these factors, interventions that effectively support the continuity of ART as HIV-infected individuals transition back to their communities are part of more comprehensive national STTR HIV prevention and intervention efforts.

The STTR approach suggests that HIV-infected justice-involved individuals (that is individuals who have been involved in the criminal justice system, such as through incarceration) could benefit from an intervention to enhance both timely and continued engagement in HIV care and adherence to ART. However, as studies that have identified barriers to care engagement and ART adherence among released HIV-infected individuals indicate, there is not one single ideal, addressable target for improvement. Instead, research shows that multiple barriers hinder HIV-infected individuals from accessing care and adhering to prescribed medications. Challenges that interfere include: returning to neighborhoods that lack social and economic capital and contain drug-using social networks [24, 25]; facing intersectional discrimination of both incarceration and HIV [24, 26]; strained interpersonal relationships [17]; mental illness and substance abuse [24, 27]; and difficulty accessing housing [17, 18, 26, 28], transportation [18, 28], insurance, and employment [17, 26, 28]. Such findings suggest the need for multi-component interventions that can address multiple factors simultaneously to successfully help HIV-infected prisoners maintain viral suppression during reentry.

### Purpose of the current study

**Fig. 1:**
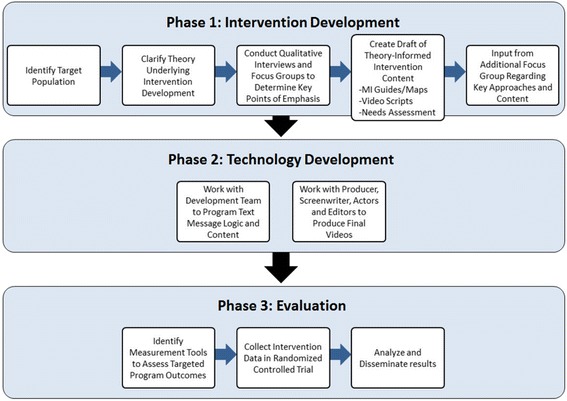
imPACT intervention development process

Our research team developed Project imPACT (individuals motivated to Participate in Adherence, Care and Treatment), a multi-component approach for HIV-Infected recently incarcerated individuals that specifically targets their care linkage, retention, and medication adherence by addressing multiple barriers to care engagement. The ultimate goals of this intervention are to improve the health of HIV-infected individuals recently released from prison and reduce HIV transmission to their communities by maintaining viral suppression. This paper describes the intervention and technology development processes, based on best practices for intervention development and process evaluation [29]. As shown in Fig. 1, this process included: 1) identifying the target population; 2) clarifying the theoretical basis for intervention design; 3) describing features of its foundational interventions; 4) conducting formative qualitative research; 5) integrating and adapting foundational interventions to create and refine intervention content based on target audience feedback. These stages are described in more detail below along with the final intervention product.

## Methods/Design

### Target population for the intervention

We designed the intervention for HIV-infected men and women who were English-speaking, age 18 years and older, incarcerated within the Texas or North Carolina state prison systems [Texas Department of Criminal Justice (TDCJ) or the North Carolina Department of Public Safety (NCDPS)], treated with ART with a recorded plasma HIV RNA level of < 400 copies/mL and expected to be released to the community within approximately 12 weeks. We elected to focus on individuals in these two states because these were settings with a strong research focus and a relatively large number of HIV-infected individuals who faced documented challenges to care engagement after release. Additionally, combined, the two states incarcerate approximately 1 in 7 of all individuals incarcerated in a U.S. state prison system in the US [30]. In each of these settings, it is standard practice for prison staff to conduct routine discharge planning before release, which is limited to the provision of referrals to community clinics, housing, and other services based on availability and need.

Given that incarcerated individuals have higher rates of HIV infection than the general population and that they face unique challenges during community reentry, we chose to design an intervention that targeted HIV-infected individuals in the two months before and three months after release from incarceration. Individuals with a suppressed viral load at the time of prison release comprised the target population for the imPACT intervention. The imPACT intervention also targets the high risk reentry period, as the target population had demonstrated an ability to adhere sufficiently to ART and care during incarceration. A lack of control of HIV, despite the structured prison environment, suggested the presence of biological or behavioral factors that would be best addressed by different interventions.

Because of the complexity of the intervention and the extensive resources that would be needed to administer each component in multiple languages, we limited this initial evaluation to English-speaking individuals with plans to adapt it for monolingual Spanish-speakers if it found to be effective. We designed imPACT for both men and women, and given that the HIV and incarceration epidemics also disproportionately affect racial and ethnic minorities [9], the research team felt that creating a intervention that could appeal to demographically diverse group of men and women, rather than targeting a particular ethnic, racial, gender or sexual orientation group, would be important for future uptake of the intervention among the populations most in need.

### Theoretical foundation

Adapting from our previous work designing motivational interviewing-based medication adherence interventions [30–36], we used a socio-ecological framework to ground the imPACT intervention in two leading health behavior theories: The Social Cognitive Theory [37] and the Information-Motivation-Behavioral (IMB) Skills model [38]. Social Cognitive Theory (SCT) posits that whether an individual successfully carries out and maintains a learned behavior is determined by the reciprocal interactions among the individual’s beliefs about his/her self-efficacy to perform the behavior, experienced responses to the behavior which generate outcome expectancies, and environmental factors that influence one’s ability to carry out the behavior [37]. The role of self-efficacy is a core tenet of SCT, such that individuals with high self-efficacy are more likely to adopt observed behaviors. Enhancing self-efficacy can increase a behavior, and mastery experiences, social modeling, and verbal encouragement can enhance self-efficacy. Hence, from SCT, a key approach to enhancing behaviors like attending clinic visits or adhering to medication is to incorporate activities that use mastery, modeling, and encouragement to enhance self-efficacy. The SCT also emphasizes the importance of reciprocal interactions of the individual with aspects of his/her environment, such as institutional or community-level barriers that HIV-infected individuals face. The IMB model asserts direct pathways between HIV-related information, motivation, and necessary behavioral skills, including medication-taking proficiency [39], as predictors of engagement and adherence. Knowledge about the medical condition including regarding available effective strategies for its management, is considered necessary but insufficient to improve behavior alone [38, 40, 41]. Motivation includes personal attitudes towards medication adherence, perceived social support for the behavior, and perceptions of how others believe people with the condition should behave. Motivation and knowledge together directly affect adherence, but also in the IMB, they act on behavior primarily through enhancing behavioral skills, particularly when the behavior is complex and involves new skills, such as with medication adherence. Information and motivation provide building blocks for the client to gain the specific behavioral tools and strategies needed to adhere. These are comprised of tactics like enlisting social support, responding to side effects, using medication reminders or other self-regulation strategies [41]. Interventions that have used the IMB model have been show to effectively improve many health-related behaviors, including antiretroviral adherence [41–43]. In the Deep South in particular, the IMB model has been shown to characterize relationships among determinants of ART adherence [44]. Being informed, socially supported, and perceiving fewer negative consequences of adherence were independently related to stronger behavioral skills for taking ART, which in turn was associated with adherence [44]. Both the SCT and IMB have demonstrated predictive validity in explaining medication and medical visit adherence [45].

**Fig. 2:**
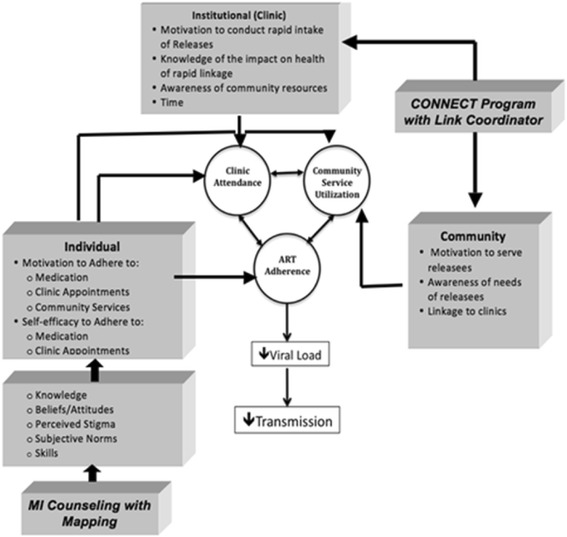
Conceptual model of imPACT intervention

Given the extensive literature demonstrating that numerous barriers to antiretroviral adherence and HIV care access occur at multiple levels of a socioecological framework, the research team integrated the IMB, SCT, and existing empirical evidence into a theoretically and empirically-grounded conceptual framework (Fig. 2) for designing the imPACT intervention (described below). The conceptual model targets both the client’s motivation and self-efficacy to adhere by providing opportunities to gain knowledge (both via interactions with a counselor and from models who are representative of the target population), clarify values, modify beliefs and attitudes, identify and address institutional and community level barriers and facilitators, and master behavioral skills, including use of medication reminders.

In addition to these health behavior theories, our intervention was informed by concepts in cognitive psychology demonstrating that graphical displays and visual representations are generally more effective in communicating complex systems of interrelated feelings, thoughts and actions, are more readily remembered, and better facilitate a shared understanding between two individuals, than language [46, 47]. Some studies have shown visual representations to be particularly useful among individuals with limited education or cognitive capacities, including among individuals involved with the criminal justice system [48].

### Role of foundational interventions

The imPACT intervention combines elements from three existing theory-based interventions our group had previously developed and tested. We elected to use a multi-component intervention based on extensive evidence of the multi-level challenges faced by formerly incarcerated individuals as well as studies demonstrating that comprehensive, multi-component medication adherence interventions are more effective than single-focus interventions [45, 49]. Given the complexity of barriers occurring at multiple levels (individual, institutional, community) of a social ecological framework for individuals released from prison both accessing medical care and adhering to ART (Fig. 2), a comprehensive approach was considered particularly imperative for the imPACT intervention. We adapted previously-developed motivational interviewing counseling interventions, integrated them with TCU cognitive mapping elements, and designed accompanying videos and electronic medication reminders primarily to influence individual-level behavior to support ART adherence and engagement and participation in care, respectively. These components acted mainly by enhancing motivation and self-efficacy to engage in these behaviors (Fig. 2), including seeking out and utilizing needed and available institutional and community resources (e.g., filling prescriptions, attending mental health or substance abuse treatment). To address institutional and community-level obstacles to accessing ART and care, we designed the imPACT intervention to integrate the fundamental components of an effective Link Coordinator program called Project CONNECT (described below). Below we describe each of the foundational interventions that we adapted and integrated to create the final imPACT intervention.

#### Motivational interviewing-based multi-component interventions for HIV-positive persons

Motivational interviewing (MI) is an effective, non-judgmental, client-centered counseling approach designed to enhance health-related behaviors [50], such as medication-taking and attending medical visits. It is grounded in SCT and allows individualized tailoring in a standardized manner, which makes it particularly well-suited to addressing complex, multidimensional behaviors. The MI counseling style is based on creating a client–counselor relationship that is a partnership, and on evidence that meeting the client where they are rather than directly confronting or directing him or her increases a client’s intrinsic motivation and self-efficacy to change behavior. MI is based on the supposition that clients feel ambivalent about unhealthy behaviors and, as such, the MI counselor guides the client toward positive behavior changes following several principles that help clients resolve their ambivalence.

In previous studies [30–35], we have successfully used multi-component, MI-based interventions for HIV-infected individuals in clinical settings to promote adherence to HIV treatment and prevention recommendations. The MI session protocols included specific steps the MI counselor followed, using a guide, to build clients’ motivation and self-efficacy, or confidence, to make changes selected, such as helping them identify strategies to overcome barriers, conducting skills-building exercises, or enhancing facilitators to healthy behavior (for example, identifying a social network member “buddy” for support, or using “reminder systems” to prompt taking medication). Scripted audio-booklet series used conversations between patient and counselor characters in an entertaining manner to prepare clients for the MI sessions, demonstrate empathy, and model mastery over behavior change. These materials are previously described in detail elsewhere [30–35]. In a two-arm, 140 participant randomized attention-controlled trial, the PACT intervention group had 2.75 times higher odds of achieving >95 % adherence than did the controls (*P* = 0.045; 95 % CI:1.023–7.398) [30]. Similarly, in a trial of nearly 500 HIV-positive participants, SafeTalk significantly reduced the number of unprotected sex acts with at-risk partners at 8 months from baseline in the intervention arm, while participants in the control arm experienced an increase in the number of unprotected sex acts [33].

Based on this success using an MI-based multi-component approach to addressing HIV treatment and prevention behavior change among patients in HIV clinical settings, we chose to adapt these programs for the in-prison and post-prison release settings.

#### TCU cognitive mapping

A cognitive map is a mental representation that humans use to acquire, categorize and store, and recall information regarding attributes of one’s physical or social environment, such as spatial relationships of places or hierarchical relationships among individuals. Cognitive mapping can be used in counseling as a multi-faceted technique to help clients spatially organize and relate ideas, feelings, and actions and to facilitate communication and problem solving in sessions. Mapping is a counselor skill similar in some aspects to clinical notetaking, and Rogerian reflective listening skills used in MI. Based on evidence that, on average, literacy levels are relatively low among prison populations [51, 52], and data regarding the myriad advantages that visual, non-text-based representations, such as cognitive mapping, offer for communicating with low literacy clients during counseling sessions, we elected to integrate this approach into existing steps in the MI-based counseling session guides. Members of our research team had demonstrated previously, with justice-involved individuals, that using TCU Cognitive Mapping Enhanced Counseling improved clients’ knowledge, confidence, and motivation regarding general HIV information, risky sex and drug use, HIV testing, and risk reduction skills [53]. These investigators and their colleagues had successfully used cognitive mapping methods within a counseling program with probationers, including those engaging in HIV-risky behaviors [54], and found that the approach was effective at increasing perceived treatment effectiveness, with a particularly beneficial impact on those with lower levels of treatment readiness. We applied these techniques to develop appropriate cognitive mapping exercises for key steps in the MI protocol (Table 1) and to train the interventionists to utilize these methods.

**Table 1:** imPACT intervention components

Intervention components	Targeted theoretical constructs	Content/components
Motivational Interviewing Sessions	• Information • Motivation • Self-efficacy • Behavioral Skills	• 2 monthly in-prison face to face sessions, with first approximately 8 weeks prior to release, augmented by: o cognitive mapping • 6 bi-weekly phone sessions after release
Accompanying Videos	• Information • Motivation • Self-efficacy	• Modeling by patient characters • Information • Motivation to take responsibility for health
Link Coordination	• Environmental Barriers	• One-time needs assessment, approximately 4 weeks prior to release • Schedule appointment at comprehensive, accessible HIV care home • Provide health care home with needs assessment results • Arrange for ADAP and drug/Medicaid assistance applications, if needed • Reschedule appointment up to two times, if missed
SMS Reminders	• Reminder	• Training in cell phone use • Assessment of medication regimen • Tailored reminder messages selected by the participant • For each ART dose due, SMS reminder message and SMS request to confirm dose taken

#### Project CONNECT--Client-Oriented New patient Navigation to Encourage Connection to Treatment

Project CONNECT is a multidisciplinary, structural, clinic-level intervention to improve linkage to HIV care [55] for recently diagnosed HIV clinic patients in the community. We elected to incorporate Project CONNECT to address the need for released inmates on ART to link quickly to HIV care before they run out of the ART they are dispensed at release, and to link to a clinic that can address the competing psychosocial barriers to care arising at the institutional and community levels. A core component of Project CONNECT is a roughly one-hour orientation visit with a social worker or facilitator within five days of the patient’s initial call to the clinic. During the orientation visit, the Project CONNECT facilitator builds rapport with the new patient. The patient completes a theory-driven semi-structured interview, a standardized questionnaire assessing psychosocial barriers to care and urgent health needs (e.g., PHQ9 to assess depression), and baseline laboratory testing. With this information, the facilitator schedules a clinic appointment within three weeks, and makes prompt referrals for substance abuse, mental health, and other ancillary services (e.g., rapid institution of prophylactic medications), as needed. In a pre-/post- trial of nearly 900 patients conducted as part of a continuous quality improvement initiative, a significantly greater percentage of the participants receiving the Project CONNECT intervention attended a primary HIV provider visit within 6 months of contacting the clinic compared to the participants from the pre-CONNECT period (81 % vs. 69 %, *p* < 0.01) [55] (http://www.cdc.gov/hiv/pdf/prs_compendium_project_connect_ei.pdf). Project CONNECT demonstrated that rapid linkage to appropriate care that systematically evaluated patients’ medical and psychosocial needs, and referred to existing resources to address specific needs, supported linkage to medical care. We incorporated a similar Link Coordinator position with Project imPACT and used the Project CONNECT model to guide us. Based on the Project CONNECT model, we designed the intervention to link individuals to a comprehensive medical home that could best provide needed services (e.g. housing referrals, substance abuse treatment, mental health treatment, etc.,) rather than have the imPACT intervention provide such services directly.

### Formative qualitative studies to inform intervention adaptation and integration

During our initial formative work, we first conducted in-depth, semi-structured interviews and focus groups in Texas and North Carolina among HIV-infected formerly incarcerated patients, and community-based HIV service providers whose clients included formerly incarcerated men and women (full details reported previously, [56, 57]). Data showed that justice-involved individuals often had a reduced sense of agency over their own lives and health after incarceration, and the importance of the system facilitating initial linkage to care after release also declined. Findings highlighted the importance of getting individuals into a medical home soon after release to address a key individual-level barrier to adherence to medication and appointments: substance abuse. Numerous community and policy level barriers that would need to be addressed included lack of housing, employment, transportation, and enrollment in safety net programs. At the same time, interpersonal and community social support were identified as key facilitators that could be built upon in an intervention at the community and institutional levels. In addition, the assemblage of barriers generated a set of competing demands and disorder in participants’ lives that made it challenging for them to attend to their health care needs consistently [57].

Health care providers (case managers, mental health care professionals, nurses, nurse practitioners, and physicians) of formerly incarcerated individuals identified similar individual, community, and organization/institutional-level obstacles to HIV care and treatment adherence and offered additional insight into the ways that these multilevel factors affect formerly incarcerated HIV-infected individuals’ abilities to engage in care and access necessary social services.

Additional barriers that providers identified included the inability of individuals who had been locked up previously to do things for themselves after not doing so during confinement, a lack of familiarity with new technology, competing demands of required reintegration activities. HIV-related stigma was discussed as causing clients to have difficulty accessing transportation to and from medical care because they feared doing so would disclose their HIV status. Providers highlighted the negative effects on health care access of poor coordination between prison and community care systems (including lack of appointment scheduling before release), as well as negative environments/social networks and lack of essential services and community resources needed to address housing, transportation or behavioral health problems, such as substance abuse.

### Key implications of formative findings

Taken together our formative studies indicated that it is essential that the imPACT intervention have an impact multiple levels to successfully engage formerly incarcerated individuals in HIV care after release. Our research team identified several specific targets that were important for the imPACT intervention to address. ART adherence challenges stemming from the chaotic nature of releasees’ lives and competing priorities confirmed the likely benefit of MI for medication-adherence skills-building and an automated medication reminder system. The need to enhance individuals’ self-efficacy and motivation to stay health confirmed the importance of including pre- and post-release MI sessions. The lack of care coordination between prison and community settings indicated the need for assisting with linkage to care immediately after release. The multiple unmet basic needs of released inmates suggested the need for a mechanism to better assess and refer these needs to appropriate community resources. The wide variability of barriers for each releasee indicated a need for individualized assessment and tailoring of strategies to achieve health goals, like that found in MI. The small supply of ART given upon release and reports of limited support from prisons for completing drug assistance and insurance paperwork prior to release, indicated the need for routine assistance completing AIDS Drug Assistance Program (ADAP) applications before release. We also developed the Link Coordination component to some extent by meeting with community clinics throughout the state to orient them to the intervention and the Link Coordinator. This socialization of the project was helpful. Clinics were those that were identified by the prison as being commonly mentioned by inmates as sources of care, and were receptive to being at receiving end of the referrals. Reports of formerly incarcerated individuals’ inconsistent experience with technology pointed to the need to provide training in the use of any technology required for participation in the intervention. And, finally, releasee experiences with stigma and discrimination that impacted their ability to engage in care, emphasized the importance of providing compassionate, non-judgmental, non-stigmatizing support through this intervention.

During the intervention development process, we conducted one additional focus group among seven HIV-infected former inmates to obtain their input regarding specific aspects of the intervention in development. Focus group members endorsed the idea that receiving support from others to facilitate the transition back to the community would greatly enhance releasees’ abilities to avoid lapses in medical care. In particular, they strongly recommended that the intervention schedule the first post-release clinic appointment for the patient. As one participant put it, “But it would have been better if they had actually made the appointment for me from prison … it would have been better than me having to get a referral that’s sent to the halfway house and allowed them to make the appointment. If the appointment had already been set up [when] I got out, then I wouldn’t have had, run out of medicine and I wouldn’t have had to wait.” Participants supported the use of videos and emphasized that the videos should indicate the need for former inmates to take ownership of their health. Participants also recommended that the intervention start before release to motivate prisoners to plan for their post-incarceration care and living situation. Participants endorsed the potential utility of cell phone reminders but stressed the need for significant training in using the technology, particularly for those incarcerated for longer stays who would be less familiar with this technology.

### Technical development of videos

To adapt the audio-booklet materials from the foundational interventions for use with prisoners, we created videos which our formative studies indicated would be more appealing and comprehensible for this population. In total, two videos were created that used the same approaches--conversations with realistic patient and counselor characters, demonstration of empathy and modeling mastery over behavior change, testimonials and individual stories--to achieve similar goals, namely introducing the intervention and enhancing participant self-efficacy and motivation to access and adhere to community-based HIV care. We worked collaboratively with The Studio, Inc. to complete the technical design, filming, editing, and final production of the videos. The Studio, Inc. is a Chapel Hill, NC production company that applies state-of-the-art techniques to the development of educational videos, including interventions aimed at health promotion and disease prevention. The Studio, Inc. team included a script writer, graphic designer, music producer, videographer, director and several actors. In collaboration with The Studio, Inc., our research group spent approximately 12 months in an iterative process developing the content, creating realistic characters, finalizing the script, filming, incorporating music and editing to produce a final product that achieved the goals of this intervention component and won a 2012 Telly Award for Best Educational Video, the premier award honoring the finest video productions. As described below, the videos were shown to individual inmates at two successive intervention visits before release.

### Technical development of SMS messaging

To update the reminder skills taught in the *PACT* intervention (prior to the rise in popularity of text messaging) to help clients overcome forgetfulness, we incorporated into imPACT a text message reminder system. This approach was consistent with the formative data we obtained, where releasees indicated they were unaccustomed to managing their own care after having spent months or years in a prison system with set schedules, and that scheduled reminders would, thus, be beneficial during the transition period. Moreover, at the time we developed imPACT we had planned to give flip-phone to trial participants to facilitate study retention and data were beginning to emerge demonstrating that text reminders could be effective for enhancing ART adherence, particularly when partnered with other intervention components [58]. We worked with computer programmers at the Cecil G. Sheps Center for Health Services Research to develop an automated, individually tailorable SMS system to deliver medication reminders. During the development process, we faced decisions regarding the type and frequency of reminders and the degree of interactivity of the SMS program. Considerations included: how often to send the messages (e.g., link them to each dose, once daily or weekly?); whether the messages should just be reminders or also be inspirational; whether to use standard or self-authored messages; how many follow-up texts per dose should be sent and at what intervals; how long after release should the texts be continued; and whether to require clients to respond to messages, and if so, using words or numbers. Because input from our formative work indicating that prisoners, particularly those who had been incarcerated for long time periods or with low literacy, would face significant challenges to using complex technology, and might find intermittent reminders confusing, we elected to send dose-based reminders that required minimal reading, writing, or interaction (Fig. 5). Because there was little evidence at the time regarding the relative advantages of standardized versus self-authored, or reminder-based versus inspirational messaging, and because our formative work indicated significant concerns regarding HIV-associated stigma and unintended serostatus disclosure, we designed the intervention to offer participants a menu of standard reminding, inspirational messages, or an option to design their own. Once the SMS system was programmed, our team conducted beta-test of the programing and made minor programming adjustments to address logical errors that came to light during testing. The final intervention is described below.

### Final imPACT intervention

**Fig. 3:**
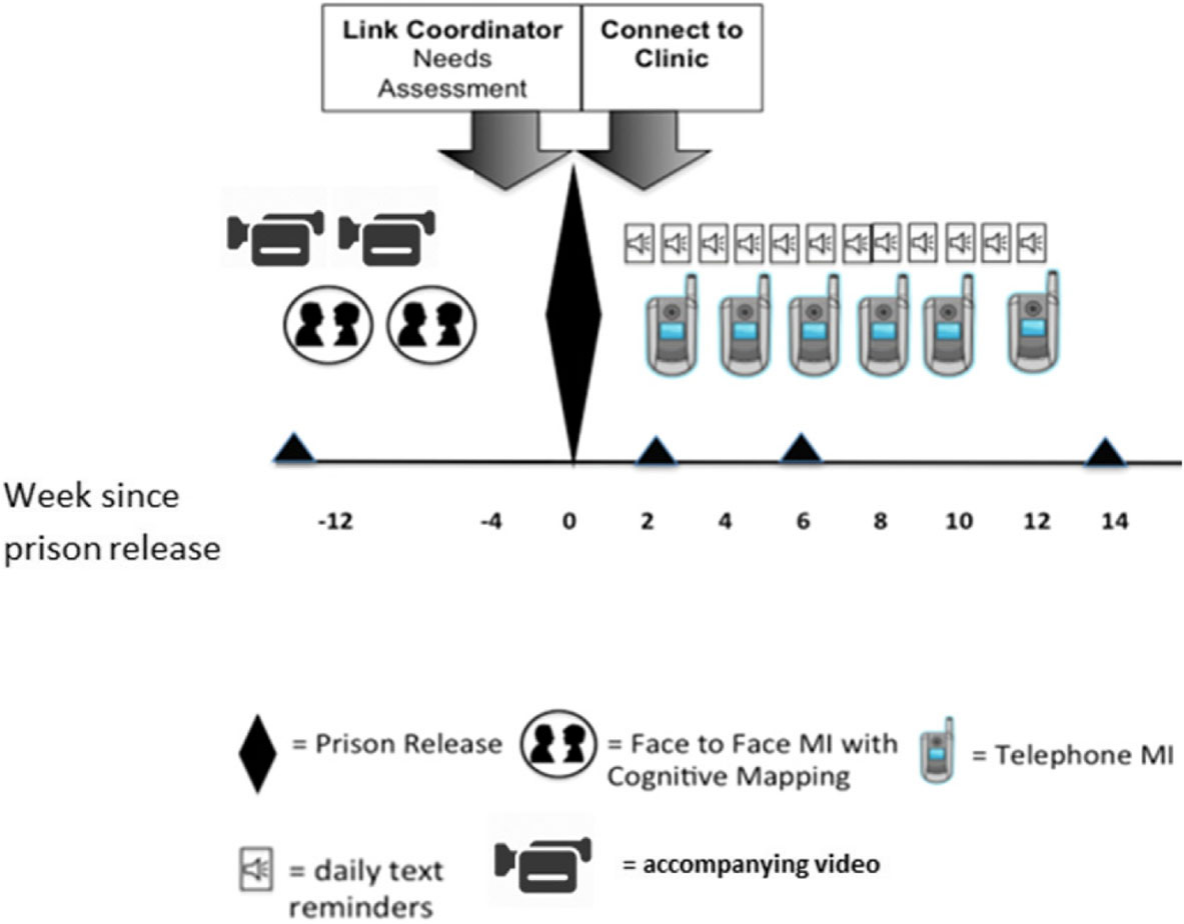
imPACT intervention components timeline

Integrating our previously developed interventions with each other and findings from our formative work, we designed the final imPACT intervention to have four main components: 1) motivational interviewing counseling augmented by cognitive mapping; 2) two relatively short videos, one to immediately precede each face-to-face in-prison MI session; 3) Link Coordination with needs assessment; and 4) medication adherence SMS reminders. Figure 3 illustrates the temporal relationship among the four components relative to each other and to the time of prison release. Below we describe each component of the final intervention.

#### Motivational interviewing (MI) augmented by cognitive mapping

imPACT MI sessions are conducted by a trained masters-level counselor in two individual face-to-face sessions in prison, approximately four weeks apart, and lasting approximately one hour each. These sessions are followed by six additional sessions delivered by telephone by the same counselor who delivers the participant’s pre-release sessions, approximately every two weeks over 12–14 weeks after release. Each session begins with building rapport and invites participants to choose from a menu of topics that are most salient to them. MI counselors use Rogerian techniques, like reflective listening, to help participants feel understood, and raise awareness of ambivalence they may feel about their chosen behavior and any discrepancies between their values and their expected behaviors. Through these techniques, counselors lead participants to make self-motivating statements to access care and adherence to ART after release. The MI counselor also uses specific techniques to build participants’ self-efficacy to make incremental realistic changes, such as helping them identify strategies to overcome barriers, build skills necessary for maintaining health, or enhance facilitators (e.g., support from family or using a medication reminder) to change.

**Fig. 4:**
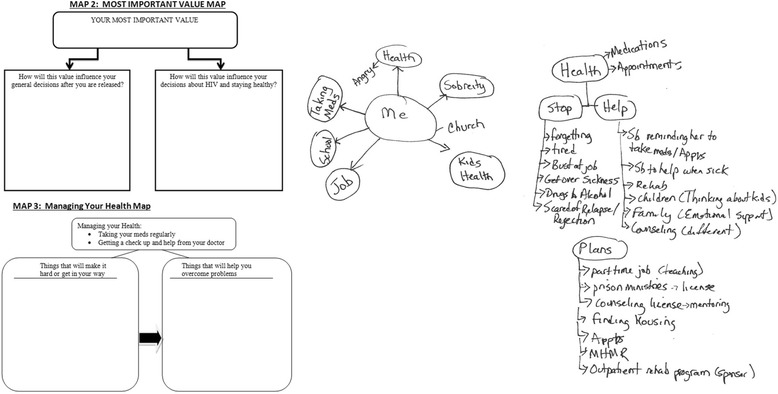
Samples of cognitive maps used in imPACT motivational interviewing session

Each in-person session is augmented by the potential use of several cognitive maps which can include both *Guide maps* which are “fill-in-the-blank” tools used to facilitate planning, decision-making, problem solving, and assessment (Fig. 4), and *Freestyle maps* which are produced “freehand” by participants in collaboration with counselors to generate brainstorming.

For each in-person and phone MI session, we designed a stepwise guide (Appendix, Table 2) for the counselor, adapted for the target population from previous MI interventions. Each session guide contains clear objectives for the session; a list of materials (e.g., copies of cognitive maps, pencils, etc.) and conditions (e.g., a private room, freedom from distractions, etc.) the counselor will need to carry out the session; and a description up to 10 steps that comprise the session. One exception to this is session 3, the first phone session which, as a transitional session, is abbreviated to four steps rather than 10. The guide provides a recommended structure that allows the counselor to flexibly address client concerns as they arise. Each step in the guide includes instructions (e.g., “Use reflective listening to client’s response) and sample statements for the counselors to direct them to carry out that step by indicating the tone and intention of the step rather than serving as a verbatim statement to be used by the counselor.

As indicated in the Appendix (Table 2), the first MI session focuses on rapport-building and unique steps that facilitate building trust and getting to know the client, such as assessing and clarifying their values and their expectations for release. In general, the subsequent MI sessions focus on preparing him/her to adhere to ART and engage in care after release, and follow seven key steps: 1) Topic Selection; 2) Assessing Facilitators & Barriers; 3) Identifying Ideas; 4) Rating Perceived Importance & Confidence to address the Selected Topic; 5) Exploring Goal Setting Based On Readiness; 6) Exploring Advantages And Disadvantages; and 7) Making Plans For Coping. Each session ends with Closure that includes summarizing the session and (except in the last session) scheduling the next session.

To maintain intervention quality and fidelity, the sessions are designed to be audio-recorded, with permission from participants, and for counselors to use a standardized written data recording sheet to record the content of each step of the session. The data recording sheets can also serve as a bulleted guide to help the counselors remember to complete each step. These sheets and the audio-recordings are intended for use during routine clinical supervision.

#### Videos

Before each in-prison MI session, participants are shown (on a private computer with headphones) one of the two 15 min videos that we produced for the trial and provided an orientation to the intervention and prepared the participant for each upcoming MI session, as described above.

#### Brief link coordination with needs assessment

Shortly before release, a study Link Coordinator meets with the participant once, and using a standardized set of questions, conducts an evaluation of anticipated needs following community re-entry. The needs assessment pays particular attention to plans for clinical care, medication access, housing, and transportation. The intervention is designed for the Link Coordinator to schedule a clinic appointment for the participant, ideally within 5 days of release, and to share the needs assessment with the referral clinic before the appointment. The goal of the needs assessment, based on the Project CONNECT model, is to link a patient with a comprehensive clinic and inform the clinic of the patient’s psychosocial and medical needs, thereby resulting in appropriate referrals for other needed services, in addition to better care engagement. In addition, the Link Coordinator submits applications for state ADAP or pharmaceutical company drug assistance programs, as needed. Following release, the Link Coordinator supports participant clinic attendance by calling participants with appointment reminders and leveraging available community resources, when necessary. All post-release Link Coordinator encounters with the participant are conducted by telephone. If the initial clinic appointment is not kept by the participant, the Link Coordinator makes one additional clinic appointment on behalf of the participant. All interactions between the Link Coordinator and the participant cease once the arranged clinic appointment is attended or, in the case of two missed appointments, after the second missed appointment.

#### Text message antiretroviral medication reminders

**Fig. 5:**
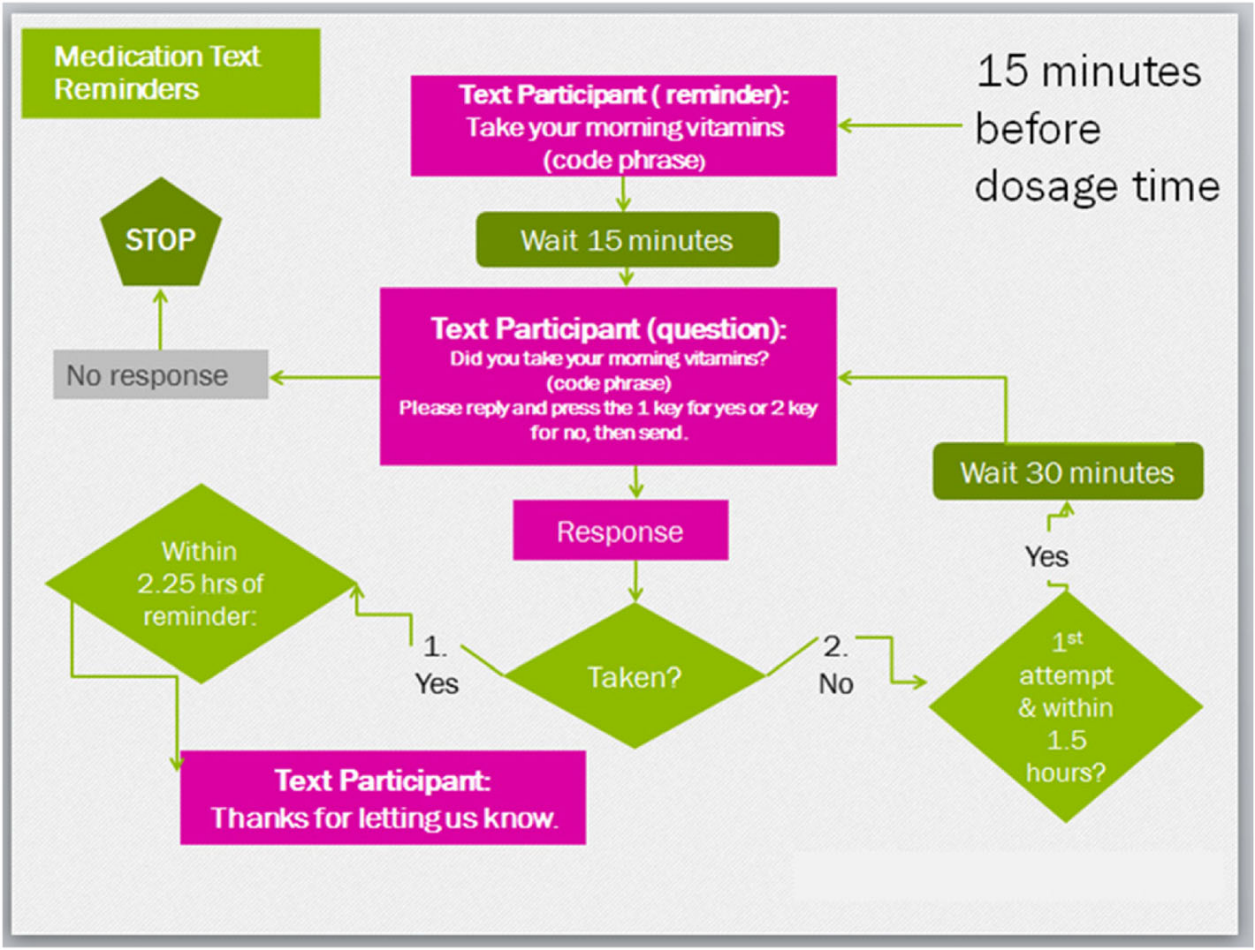
Text message logic flow diagram

In this component of the intervention, to support adherence, participants receive medication reminder text messages on study-provided flip phones 15 min before each scheduled ART dose for the first 12 weeks post-release. Text messages consist of phrases created by participants, after study staff provides examples (e.g., “Remember to take your vitamins”). As shown in Fig. 5, the timing of text reminders is customized to the participant’s regimen, which the Link Coordinator assesses prior to release, and are followed in 15 min by a query text asking if they have followed through with the action prompted by the code phrase (e.g., “Did you take your vitamins?”), and instructions to press 1 for ‘yes’ and 2 for ‘no.’ When the response is ‘yes’ and occurs within 2 h of the dose time, the participant receives a text message that says, “Thanks for letting us know.” The system does not respond to a participant’s response if it is received more than 2 h after his/her dosage time, to avoid encouraging participants to take their ART more than 2 h after the dose is due. When the response is ‘no’ and is sent within 1.5 h, the following text is sent: “Thanks for letting us know. We will check back in 30 min.” If an additional ‘no” response is received within 2.25 h of the reminder, the participant is sent the following text message: “Thanks for letting us know.” No further texts are sent to those not responding to the initial question until the next scheduled dose and reminder.

## Discussion

This article describes the development of imPACT, a multi-component intervention for linkage and engagement in HIV medical care for prisoners during their transition from incarceration to community reentry. Based on a literature review, qualitative research with men and women from the target population and care providers, integration of proven interventions and behavioral theory, the final imPACT intervention focused on the transition period two to three months before and three months after prison release. It emphasized pre-release readiness, pre- and post-release supportive non-judgmental counseling, linking individuals to a HIV care clinic within five days of release and providing technological supports through videos and text messages. Although linkage and adherence to community-based care and treatment are recognized as critical both to the health of HIV-infected individuals and to HIV prevention in their communities, particularly among recently released prisoners, few interventions specifically target the required skills and means to overcome community-level barriers in this population. We developed Project imPACT to fill this gap.

The imPACT intervention is grounded in psychological and health behavior change theories [37, 41] and informed by qualitative interviews and focus group discussions as well as direct feedback from a focus group of HIV-infected formerly incarcerated individuals. The final intervention includes two videos, motivational interviewing counseling, link coordination and text message reminders. The final videos use patient characters to role model how similar others have maintained health successfully, including motivation and skills that helped them after release. Motivational interviewing counseling sessions help participants identify internal motivations for staying healthy and develop skills and self-efficacy to overcome barriers, using features known to enhance intervention effectiveness, such as elicit-provide-elicit techniques [50] and cognitive mapping [46, 48, 53, 54]. Link coordination serves to overcome community and institutional barriers to accessing care.

The current article provides a useful model for how researchers can develop, test, and refine multi-component interventions to address HIV care linkage, retention and adherence. The HIV prevention field that attempts to reduce HIV spread via enhancing the HIV treatment cascade is still relatively new but rapidly growing [5, 7, 10], and, while there is a call to develop interventions to simultaneously tackle multiple steps in the cascade [10], there is currently a lack of consensus on a model for doing so. Our development approach had several strengths, including the use of multiple theories and a novel focus on both linkage and adherence; adaptation of previously developed interventions via significant target audience input; an iterative approach to development and usability testing where the intervention was revised at several points in the process; and the application of state-of-the-art video and text-messaging technology.

We are just completing the evaluation of the efficacy of the imPACT intervention to help patients maintain a suppressed viral load for 24 weeks after prison release in a randomized controlled trial (Fig. 1) conducted from March 2012 through February 2015 (RCT; Clinical Trials registration number NCT01629316) [59]. Participants are 381 HIV-infected participants receiving HIV care in the Texas or North Carolina state prison systems who were virally suppressed in prison and within 3 months of release from prison. Our evaluation will include analysis of process data to assess the acceptability, feasibility, and usefulness of the intervention from the perspective of study participants, cost of intervention delivery, effects on adherence and clinic attendance.

## Appendix 1

**Table 2:** imPACT motivational interviewing sessions’ objectives, steps, visual aids, and sample content

Face-to-Face In Prison imPACT Motivational Interviewing Sessions		
Session/Step	Maps/Visual Aids used in Step	Examples of Sample Statements Provided to Counselor in the Guide for that Step
Session 1 Objectives		
1. Build rapport with client 2. Explore general post-release expectations		3. Explore post-release expectations for ART adherence and treatment 4. Develop an action plan for time between Session 1 & 2
Step One: Introduction	No Map	The most important thing for you to know before we start is that this is a time for you to explore any concerns that you have about taking your HIV medicines or getting treatment, especially what these things will be like for you when you get released from prison.
Step Two: Discussing What Is Important To You.	Map 1: “What is Important to You?” Map	I’d like to spend some time learning a bit about you and about the things in life that matter to you. Looking at this worksheet called “What is Important to You” in different areas of your life, You are in the center. Let’s look at different areas starting with your Family and Friends.
Step Three: What Makes Me Tick	A Matrix of Values with Pictorial Icons	If it sounds alright with you, I’d like to go through a list of values that are important to some people—we call this list “What Makes Me Tick”.
Step Four: Exploration Of Choices And Values	Map 2: Most Important Value Map	Tell me about how [MOST IMPORTANT VALUE] influences your decisions now. Response and Reflection
Step Five: Adherence & Hiv Treatment Assessment	Map 2: Most Important Value Map	Tell me a bit about how [MOST IMPORTANT VALUE] affects the decisions you make about your HIV treatment and staying healthy. Response and Reflection
Step Six: Explore Release Expectations	Map 3: Managing Your Health Map	I’m wondering what you think might help you stay healthy and take your medicine after you get out? [Use “MAP 3: MANAGING YOUR HEALTH MAP” to explore barriers and facilitators to post-release engagement in medical care and medication adherence.]
Step Seven: Making Plans	Map 4: Before the Second Session Map	If it’s OK, let’s think together and fill out this map (4) about some things you would like to do before your next session. What steps can you begin now to prepare for success after release? Of the ideas that we’ve talked about, what specific things do you feel ready to try?
Step Eight: Summarize Session	No Map	Thank you for sharing all of this information with me. I know that sometimes it’s not always easy to think about these things, and I appreciate you being so open. Now I have a better understanding about what you think your life might be like after you get out of prison, such as [summarize facilitators and barriers, referencing Managing Your Health Map].
Step Nine: Closure	Map 4	We’ll be meeting again in about a month, and we’ll have more time to talk about how you’re feeling thinking about what your life might be like after you get out then.
Session 2 Objectives		
1. Continue to build rapport with client 2. Review Session 1 3. Update progress on “Before Second Session Map”		4. Complete Link Coordinator Referral Form 5. Identify Support Person 6. Prepare for 1^st^ Phone Counseling Session
Step One: Introduction	Map 3: Managing Your Health Map	The last time we met we spent some time talking about things that are important to you. You mentioned [VALUES CHOSEN IN SESSION 1] as things that you think about when you make decisions about your health. Let’s review the two Maps we created together …
	Map 4: Before the Second Session Map	
Step Two: Topic Selection	A Matrix of HIV Care Related Topics with Pictorial Icons	Again, these sessions are a time for you to talk openly without being judged, so what, if any, topic on the list would you like to talk about today? I’d like to understand a little more about what [SELECTED TOPIC] means to you in terms of your HIV treatment
Step Three: Assess Facilitators & Barriers	Map 3: Managing Your Health	I’d like to understand more about how you are feeling about [PROPOSED BEHAVIOR/TOPIC]. Let’s think about what it will be like for you after you are released: Do you think [PROPOSED BEHAVIOR] will be something you are able to do all, most, some or none of the time? [REFER TO MANAGING YOUR HEALTH MAP FROM SESSION I] What do you think may help you to [PROPOSED BEHAVIOR] after you get out? What do you think may get in the way? Reflection
Step Four: Identify Ideas Using Elicit-Provide-Elicit	Map 5: Generating Ideas for Success	1. Elicit ideas: Have you had any previous experience trying to do [SELECTED BEHAVIOR]? 2. Provide more information: Would you like to talk about some of the ideas you saw in the video or other ideas I’ve heard that have worked for other people? 3. Elicit their reaction: Do any of these ideas sound like something you’d like to try?
Step Five: Rate Importance & Confidence	Visual Analogue Ruler	To help me understand exactly how [important this is to you/confident you are you can do this], on a scale from 0 to 10 with 0 being not at all [important/confident] and 10 being very [important/confident], how important is [PROPOSED BEHAVIOR] to you? Question down: Why an [stated number] and not a [number 2–4 less than stated number]? Question up: Why an [stated number] and not an [number 2–4 more than stated number]? What would it take for you to move from a [stated number] to a [number 2–4 more than stated number]?
Step Six: Explore Goal Setting Based On Readiness	None	Ready: It sounds like you may be ready to think about trying to take some/a small step(s) toward [PROPOSED BEHAVIOR] after you are released. Is that right?
Ready = Moderate/High In Importance And Confidence		Not Ready: What would it take for you to be ready to take a small step towards [PROPOSED BEHAVIOR] between now and the next time I speak with you after you are released?
Not Ready = Very Low In Importance And Confidence		
		Possibly Ready: It sounds like you value moving towards [PROPOSED BEHAVIOR] but you are not quite ready. What would it take for you to get ready? OR What would it take for you to make this step between now and the next time I speak with you after you are released?
Possibly Ready = All Others		
Step Seven: Exploring Advantages And Disadvantages	None	Can you tell me some things you might like about [PROPOSED BEHAVIOR]? What about some things you might not like about [PROPOSED BEHAVIOR]? Where does this leave you now? [Provide double-sided summary]
Step Eight: Link Coordinator Referral Form	Referral Needs Assessment Form	I will not share anything you’re not comfortable with. But if it’s okay with you, I’d like for us to work together to jot down some notes to share with the Link Coordinator.
Step Nine: Identifying Support Person	None	Tell me about the people you think will be in your life on the outside, and who might give you the support you need to meet your goals for being healthy.
Step Ten: Making Plans For Coping	Matrix of Coping Strategies with Icons	Thanks for working with me to come up with a plan for when you’re released from prison. One thing I’d like to talk about now, if it’s okay, is what might happen if your plan doesn’t work out.
Step Eleven: Closure		
Phone MI Sessions after Prison Release		
Session 3 Objectives		
1. Reconnecting with participant 2. Inform participant about upcoming phone counseling sessions 3. Assess how participant has been dealing with release		4. Check in to see if participant has attended doctor’s appointments 5. Assess Release Plan with Link Coordinator 6. Prepare for next 2 week plan to initiate or maintain care and treatment
Step	Examples of Sample Statements Provided to Counselor in the Guide for that Step	
Step One: Introduction	The last time we met we spent some time creating an action plan for after you were released. What do you remember about your action plan?	
Step Two: Doctors Visit	Now I would like to talk about your Doctor’s visit. How was your appointment with [NAME OF DOCTOR OR CLINIC]? How much do you feel you have what you need to stay healthy?	
Step Three: Medication Adherence	Finally, if it is ok with you I would like to check in to see how things are going for you with taking your meds?	
Step Four: Closure	Review session and any changes to action plan	
Sessions 4–8		
1. Reconnecting with client 2. Review previous session 3. Assess progress with adherence goals		4. Make Plans for Coping 5. Prepare for next 2 week plan to improve or continue adherence plan [not included in session 8) 6. Prepare for closure from the intervention/transition [sessions 7 and 8 only]
Step One: Greeting & Review Of Phone Session Protocol		
Step Two: Review Previous Session		
Step Three: Topic Selection		
Step Four: Assess Facilitators & Barriers		
Step Five: Identify Ideas to Overcome Barriers and Enhance Facilitators		
Step Six: Rate Importance & Confidence to Use Ideas to Address selected Topic		
Step Seven: Explore Goal Setting		
Step Eight: Exploring Advantages And Disadvantages		
Step Nine: Making Plans For Coping		
Step Ten: Closure		

## Abbreviations

imPACT: Individuals motivated to participate in adherence, care, and treatment
STTR: Seek, test, treat and retain
ART: Antiretroviral therapy
HIV: Human immunodeficiency virus
TDCJ: Texas Department of Criminal Justice
NCDPS: North Carolina Department of Public Safety
RNA: Ribonucleic ACID
U.S.: United States
IMB: Information, motivation, behavior
SCT: Social cognitive theory
MI: Motivational interviewing
PACT: Participating and communicating together
TCU: Texas Christian University
Project CONNECT: Client-oriented new patient navigation to encourage connection to treatment
AIDS: Acquired immunodeficiency program
ADAP: AIDS drug assistance program
